# Supplementary material for: Induced pluripotent stem cell derived pericytes respond to mediators of proliferation and contractility
Source: Stem Cell Res Ther. 2024 Mar 3;15:59. doi: 10.1186/s13287-024-03671-x (PMC10910734; doi:10.1186/s13287-024-03671-x)
Supplement: Supplementary file 1 — Additional file 1: Supplementary methods and figures. [file 13287_2024_3671_MOESM1_ESM.pdf]

SUPPLEMENTARY INFORMATION

Induced pluripotent stem cell derived pericytes respond to mediators of proliferation and contractility

Natalie E. King[1], Jo-Maree Courtney[1], Lachlan S. Brown[1], Alastair J. Fortune[2], Nicholas B. Blackburn[2], Jessica L. Fletcher[2], Jake M. Cashion[1], Jana Talbot[3], Alice Pébay[4,5], Alex W. Hewitt[1,2,4,5,6], Gary P. Morris[1], Kaylene M. Young[2], Anthony L. Cook[3], Brad A. Sutherland[1]

1. Tasmanian School of Medicine, College of Health and Medicine, The University of Tasmania

2. Menzies Institute for Medical Research, The University of Tasmania, Hobart, Australia

3. Wicking Dementia Education and Research Centre, College of Health and Medicine, The University of Tasmania

4. Department of Anatomy and Physiology, The University of Melbourne

5. Department of Surgery, Royal Melbourne Hospital, The University of Melbourne

6. Centre for Eye Research Australia, Royal Victorian Eye and Ear Hospital

*Corresponding author:

A/Prof. Brad A. Sutherland, brad.sutherland@utas.edu.au

Level 4, Medical Sciences Precinct, Tasmanian School of Medicine, University of Tasmania, Hobart, TAS 7000, Australia

Supplementary Methods

Pericyte morphology throughout differentiation

Brightfield microscopy images of neural crest and mesoderm iPericytes were captured 10x on a Zeiss microscope using a AxioCam ICc5 camera (Imbros) at days 1, 4, 7, and 10 of differentiation and at passage 2 (P2) after differentiation.

Pericyte morphology analysis

Phase contrast micrographs of neural crest and mesoderm iPericytes were captured with the 4x objective on a Zeiss microscope using a Camera AxioCam ICc5 (Imbros). Each individual pericyte was manually assigned to a morphological subtype based on their appearance according to the criteria outlined previously (1). Percentage of each morphology within cultures was calculated from N = 746 (neural crest iPericytes) and 759 (mesoderm iPericytes) cells analysed across one culture for each differentiation method.

Pericyte expression analysis

To estimate the percentage of pericytes that expressed PDGFRβ and CD13, fluorescent images were captured with the 20x objective on an Olympus FV3000 Super Resolution confocal laser scanning microscope (Olympus, Japan) with identical settings for each marker between HBVPs, mesoderm iPericytes, and neural crest iPericytes. Images were processed in ImageJ using despeckle, then B&C manually thresholded for HBVPs until cellular autofluorescence was minimal. B&C thresholds were propagated to mesoderm and neural crest iPericyte images, then cells were manually assigned positive expression if cytosolic (CD13) labelling and cytosolic plus puncta (PDGFRβ) labelling were present above threshold

in each DAPI positive cell, or negative if labelling was not present. A minimum of 250 DAPI positive cells was counted for each group.

RNA Sequencing and differential gene expression analysis

Comparison of neural crest iPericytes to mesoderm iPericytes

Following library preparation for each sample, RNA sequencing (100bp single-end run) was carried out using an Illumina NovaSeq platform. Following demultiplexing and quality control, the cleaned sequence reads were aligned against the *Homo sapiens* genome (build version hg38) using the STAR aligner (v2.7.10a) (9). Raw gene counts were generated and transcripts were assembled with the StringTie tool (v2.1.4) (10). Differential gene expression analysis was conducted using DESeq2 (v1.36.0) (11) in R (v4.2.1) with an adjusted p-value (false discovery rate) <0.05 and absolute log2(fold-change) > 1 considered to be differentially expressed. Gene ontology enrichment analysis was conducted using clusterProfiler (v4.4.4) (12).

Comparison of three iPSC lines differentiated to three mesoderm iPericyte lines

Following library preparation for each sample, RNA was sequenced to a minimum depth of 20 million reads with 150 base paired end lengths. Raw sequencing data was processed with TrimGalore (v0.6.7) (2). The sequencing adapter (CTGTCTCTTATACACATCT) was trimmed from each read and 1 base pair was removed from the 5' end of each read. A read quality Phred score threshold of 30 was used to remove low quality reads. Reads with a length of less than 25 base pairs after trimming were discarded. The quality of the sequencing data was then evaluated using FastQC (v.0.11.9) (3), and all data met the necessary quality metrics for analysis.

Gene expression was quantified using Salmon (v.1.8.0)(4). The Salmon indexed transcriptome for *Homo sapiens* (GRCh38) was downloaded using RefGenie (v.0.12.1) (5) and Salmon used to quantify gene expression against this transcriptome in mapping-based mode. The sequencing library type was ISR. Within Salmon, --validateMappings was employed to improve the sensitivity and specificity of the read mapping (and thus quantification accuracy); --seqBias and --gcBias were employed to enable Salmon to learn and correct for sequence-specific biases and fragment-level GC biases respectively. The result of this analysis was per sample quantification of transcript expression.

Differential gene expression analysis was performed using DESeq2 (v1.34.0) (6) to compare gene expression between iPSCs and mesoderm-iPericytes. Tximport (v1.27.1) (7) was used to import transcript-level abundance into R (v.4.3.0) and summarise abundance to the gene-level. Genes with less than 1 read across each group were filtered out. Heatmaps were generated using pheatmap (v.1.0.12) (8).

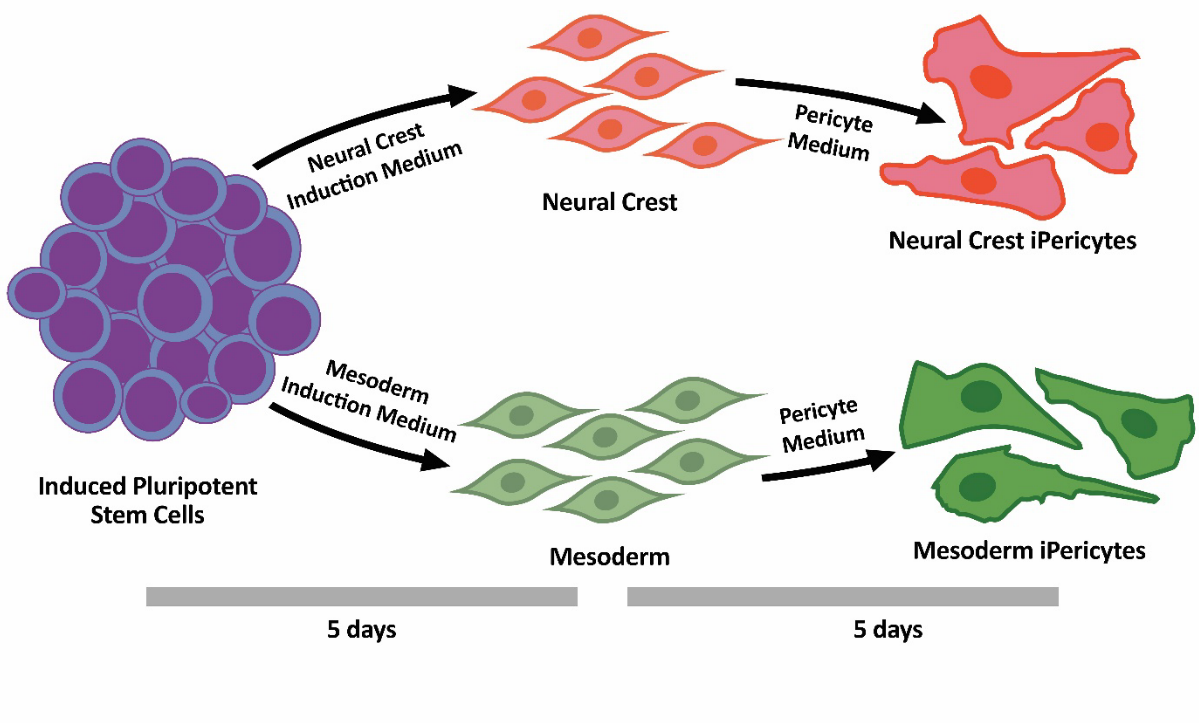

Figure S1. iPSCs can be differentiated into pericytes through the neural crest and mesoderm induction pathways. iPSCs were placed in either mesoderm induction media or neural crest induction media for 5 days. Media was then replaced with complete pericyte media for another 5 days to generate neural crest iPericytes or mesoderm iPericytes. Protocol adapted from Faal et al. (13).

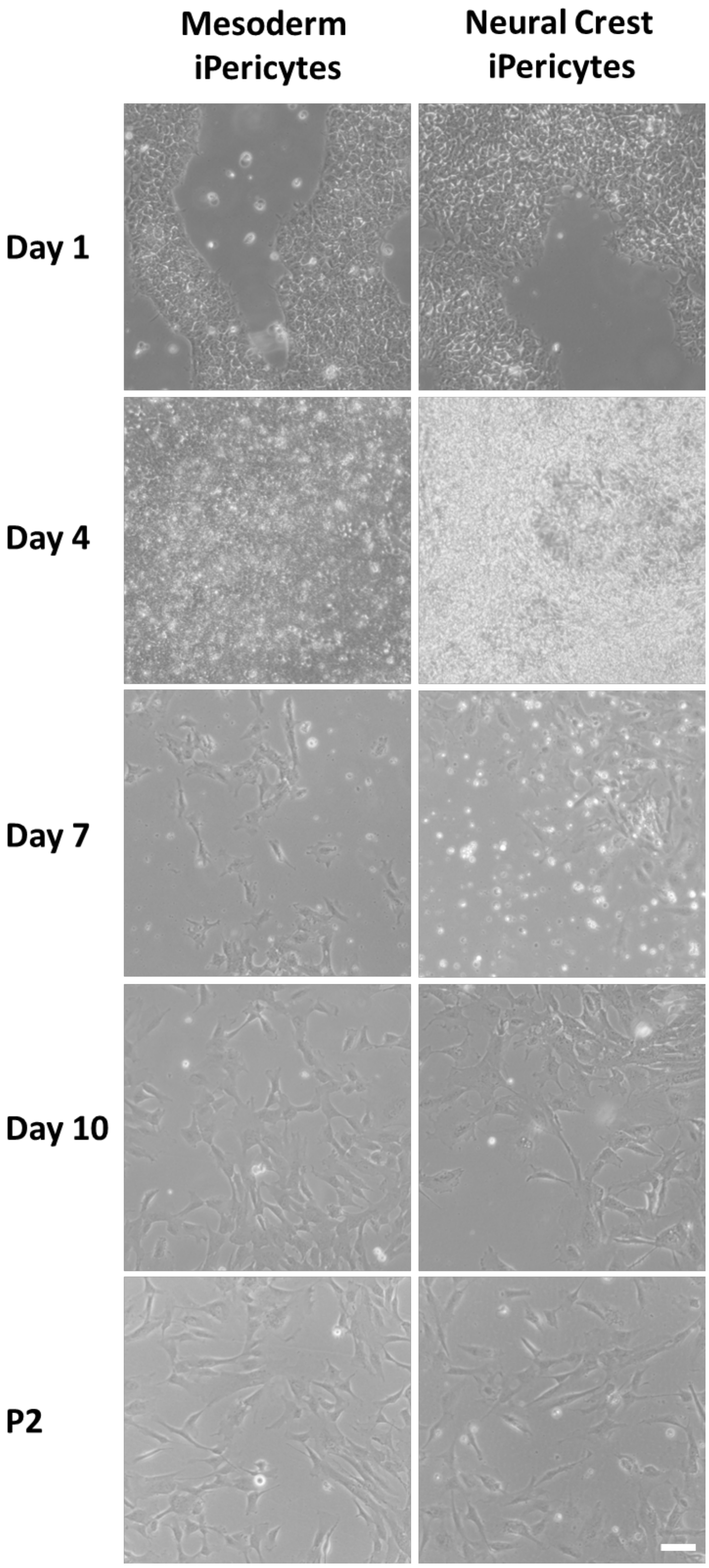

Figure S2. iPericytes display similar morphology irrespective of lineage throughout differentiation. Brightfield microscopy images were taken at days 1, 4, 7, and 10 of differentiation and at passage 2 (P2) after differentiation. Scale = 50 µm.

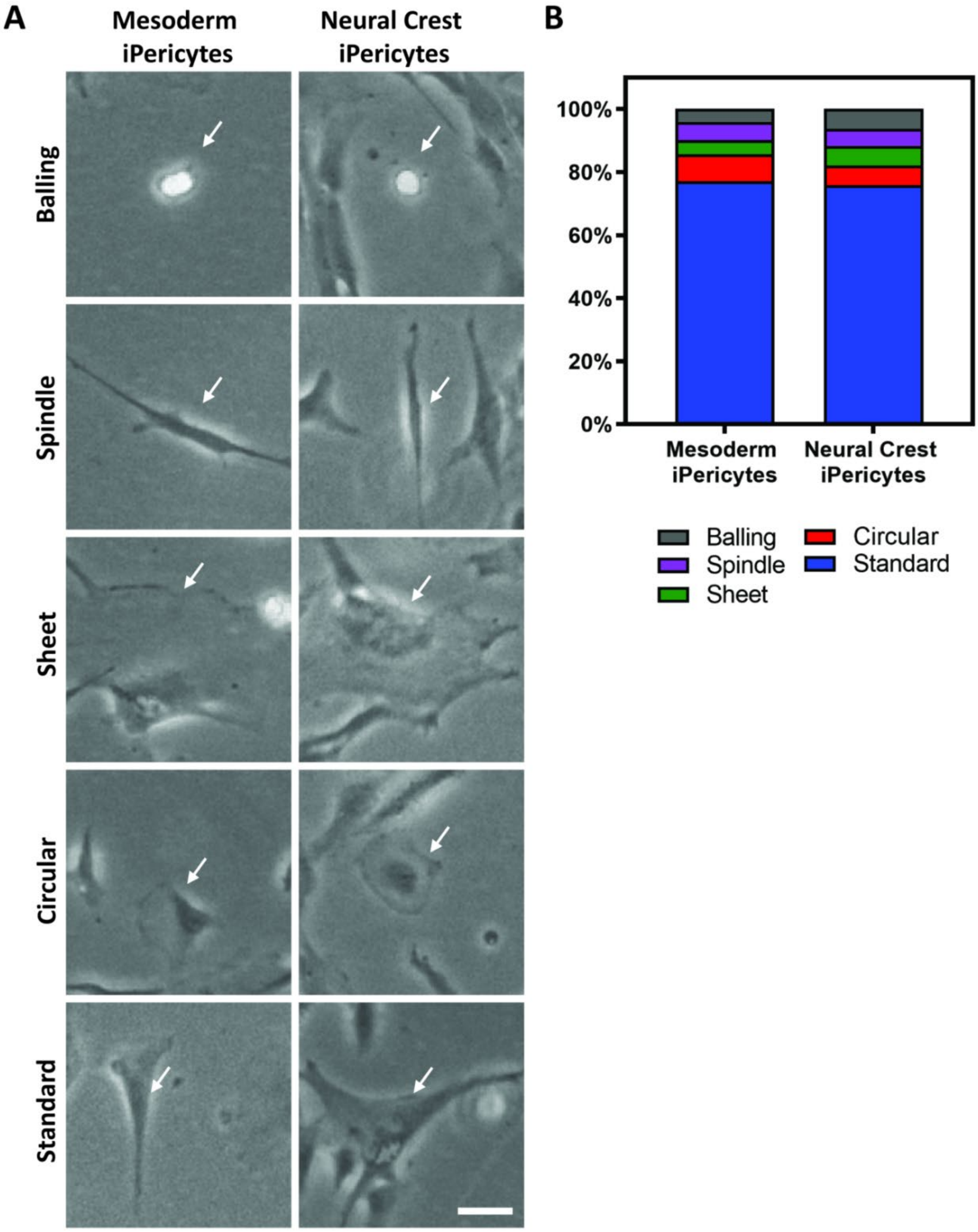

Figure S3. iPericytes display heterogenous morphology consistent with HBVP cells *in vitro.* Pericyte morphology was classified following the criteria outlined in Brown et al. (1). (A) Both neural crest iPericytes and mesoderm iPericytes display the five key morphological classifications of pericytes *in vitro*: balling, spindle, sheet, circular, and standard. (B) Quantification of the percentage of each pericyte morphology subtype in neural crest iPericyte culture (746 cells) and mesoderm iPericyte culture (759 cells). Scale = 20 µm.

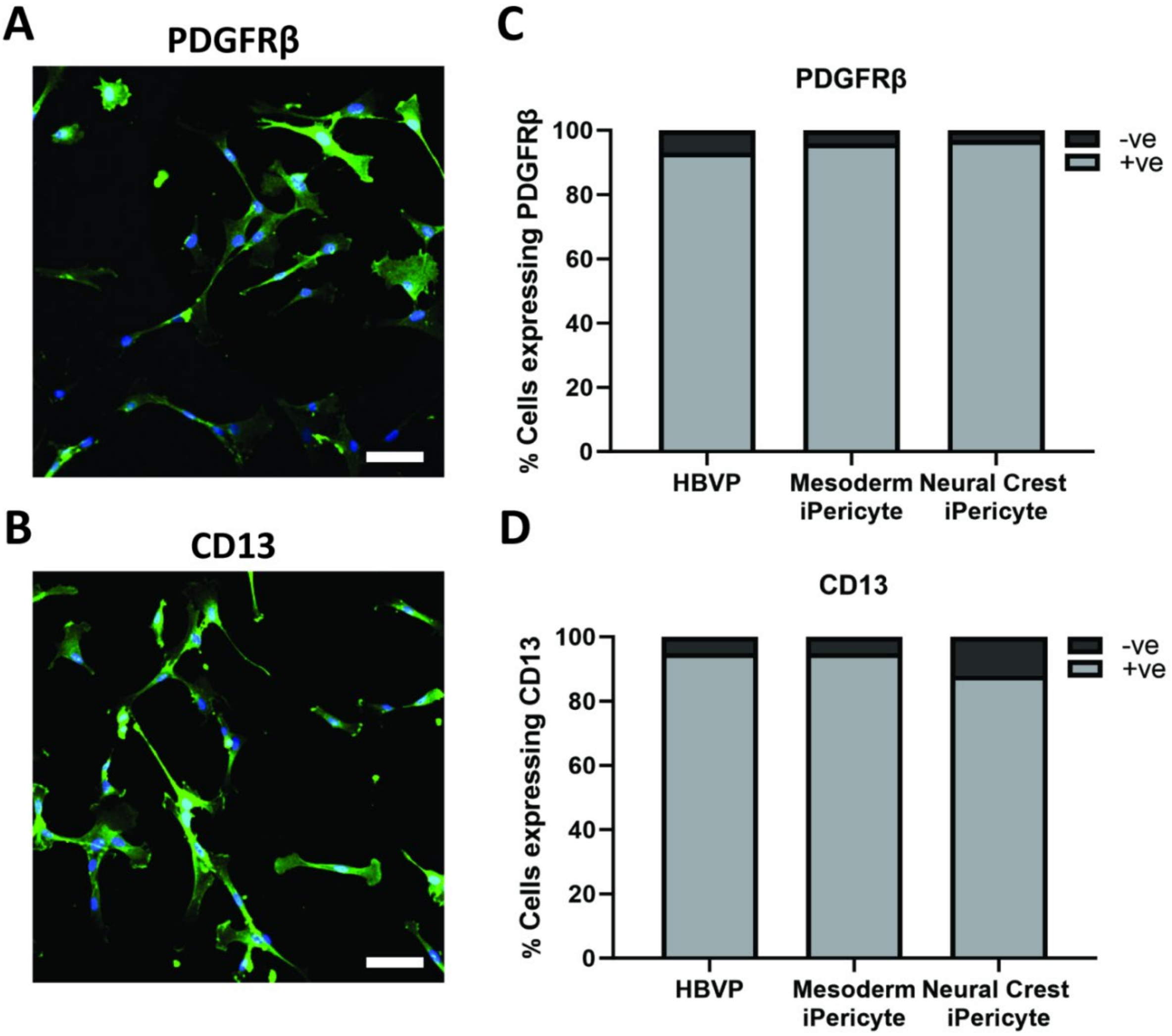

Figure S4. Quantification of percentage of cells expressing PDGFRβ and CD13. Fluorescent image of HBVPs labelled for (A) PDGFRβ or (B) CD13 and DAPI (blue). Scale = 10 µm. Quantification of percentage of pericytes positive (+ve) or negative (-ve) for (C) PDGFRβ (individual cells analysed = HBVP: 323; mesoderm iPericyte: 344; neural crest iPericyte: 403) and (D) CD13 (individual cells analysed = HBVP: 265; mesoderm iPericyte: 261; neural crest iPericyte: 302) following immunocytochemistry.

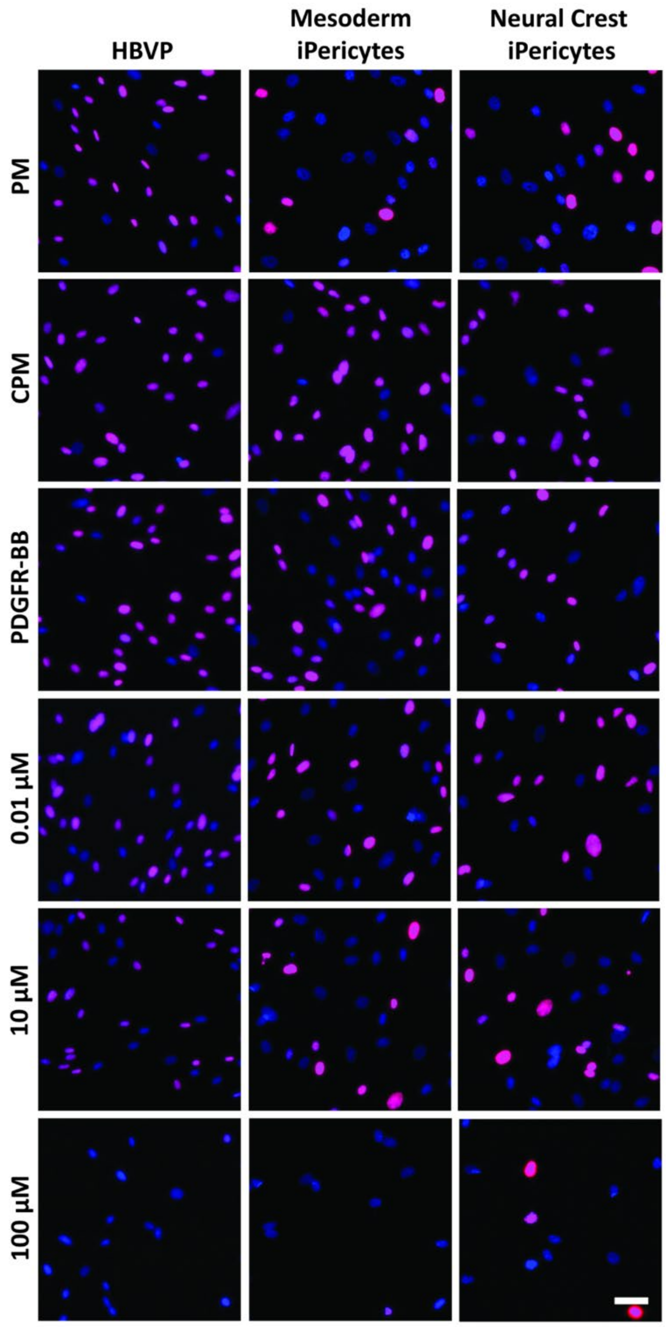

Figure S5. PDGF-BB and imatinib alter proliferative cell number of iPericytes *in vitro.* Representative images of EdU (magenta) staining in HBVP, mesoderm iPericytes and neural crest iPericytes treated with basal pericyte media (PM), complete pericyte media (CPM), PM + PDGF-BB (a pericyte growth factor), and PM + PDGF-BB + increasing concentrations of imatinib (a PDGFRβ inhibitor). Total cells were identified using DAPI (blue). Scale = 50µm.

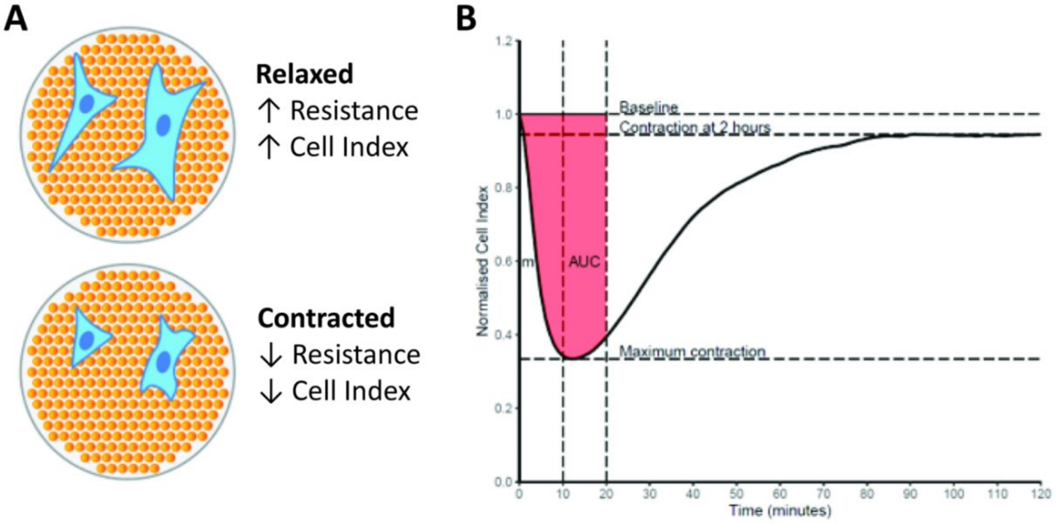

Figure S6. Schematic of pericyte contraction measurement and analysis using the xCelligence system. To test contractile function of neural crest iPericytes and mesoderm iPericytes in comparison to HBVPs, the contractile response of cells was measured using an xCelligence system. (A) Cells were plated in specialised cell culture plates with electrodes which measure electron flow. Relaxed cells have greater cell volume meaning larger impedance to electron flow, thus increased resistance, which is interpreted as a higher 'Cell Index'. Contracted cells have reduced cell volume meaning less impedance to electron flow resulting in reduced resistance, which is interpreted as lower 'Cell Index'. (B) For contractile studies, cell index for each condition was measured every minute for 2h and was normalised to time point 0, when vasoactive agents were added. To quantify differences between groups, three measures were extracted from the normalised cell index over time: slope (*m*) shows the rate of contraction over the first 10 mins and is calculated as the slope of the linear equation $y = mx+b$ for each replicate from $t = 0$ to $t = 10$; area under the curve (AUC) shows the cumulative change in contraction over time and is calculated as the area (A) under the curve between $t = 0$ and $t = 20$ mins; Δ cell index shows the change in cell index from baseline at specific time points, e.g. at the time of maximum contraction, contraction after 2h.

Supplementary Data

The below is code used in QuPath for the analysis of the proliferation assay.

Select all ROI. groovy

```
createSelectAllObject(true);
```

EDU Analysis. groovy

```
createSelectAllObject(true);

selectAnnotations();

runPlugin('qupath.imagej.detect.cells.PositiveCellDetection', '"detectionImage": "DAPI",
"requestedPixelSizeMicrons": 0.25,  "backgroundRadiusMicrons": 8.0,
"medianRadiusMicrons": 0.0,  "sigmaMicrons": 5.0,  "minAreaMicrons": 50.0,
"maxAreaMicrons": 600.0,  "threshold": 20.0,  "watershedPostProcess": true,
"cellExpansionMicrons": 0.0,  "includeNuclei": true,  "smoothBoundaries": true,
"makeMeasurements": true,  "thresholdCompartment": "Nucleus: EDU mean",
"thresholdPositive1": 2000.0,  "thresholdPositive2": 0.0,  "thresholdPositive3": 0.0,
"singleThreshold": true');
```

References

1. Brown LS, King NE, Courtney JM, Gasperini RJ, Foa L, Howells DW, et al. Brain pericytes in culture display diverse morphological and functional phenotypes. Cell Biol Toxicol. 2023.

2. Krueger F. JF, Ewels P., Afyounian E., Weinstein M., Schuster-Boeckler B., Hulselmans G., & sclamons. FelixKrueger/TrimGalore: v0.6.10 - add default decompression path (0.6.10). . Zenodo2023.

3. Andrews S. FastQC: a quality control tool for high throughput sequence data. 2010.

4. Patro R, Duggal G, Love MI, Irizarry RA, Kingsford C. Salmon provides fast and bias-aware quantification of transcript expression. Nature Methods. 2017;14(4):417-9.

5. Stolarczyk M, Reuter VP, Smith JP, Magee NE, Sheffield NC. Refgenie: a reference genome resource manager. Gigascience. 2020;9(2).

6. Miners JS, Palmer JC, Love S. Pathophysiology of Hypoperfusion of the Precuneus in Early Alzheimer's Disease. Brain Pathol. 2016;26(4):533-41.

7. Soneson C, Love MI, Robinson MD. Differential analyses for RNA-seq: transcript-level estimates improve gene-level inferences. F1000Res. 2015;4:1521.

8. Kolde R. pheatmap: Pretty Heatmaps. R package. https://CRANR-projectorg/package=pheatmap. 2019.

9. Dobin A, Davis CA, Schlesinger F, Drenkow J, Zaleski C, Jha S, et al. STAR: ultrafast universal RNA-seq aligner. Bioinformatics. 2013;29(1):15-21.

10. Pertea M, Pertea GM, Antonescu CM, Chang T-C, Mendell JT, Salzberg SL. StringTie enables improved reconstruction of a transcriptome from RNA-seq reads. Nature Biotechnology. 2015;33(3):290-5.

11. Love MI, Huber W, Anders S. Moderated estimation of fold change and dispersion for RNA-seq data with DESeq2. Genome Biology. 2014;15(12):550.

12. Wu T, Hu E, Xu S, Chen M, Guo P, Dai Z, et al. clusterProfiler 4.0: A universal enrichment tool for interpreting omics data. Innovation (Camb). 2021;2(3):100141.

13. Faal T, Phan DTT, Davtyan H, Scarfone VM, Varady E, Blurton-Jones M, et al. Induction of Mesoderm and Neural Crest-Derived Pericytes from Human Pluripotent Stem Cells to Study Blood-Brain Barrier Interactions. Stem Cell Reports. 2019;12(3):451-60.
